# Passive Radar Tracking in Clutter Using Range and Range-Rate Measurements

**DOI:** 10.3390/s23125451

**Published:** 2023-06-08

**Authors:** Asma Asif, Sithamparanathan Kandeepan, Robin J. Evans

**Affiliations:** 1 School of Engineering, RMIT University, Melbourne 3000, Australia; s3619211@student.rmit.edu.au; 2 Department of Electrical and Electronic Engineering, The University of Melbourne, Parkville 3010, Australia; robinje@unimelb.edu.au

**Keywords:** passive radars, unscented Kalman filter, extended Kalman filter, probabilistic data association, bias

## Abstract

Passive bistatic radar research is essential for accurate 3D target tracking, especially in the presence of missing or low-quality bearing information. Traditional extended Kalman filter (EKF) methods often introduce bias in such scenarios. To overcome this limitation, we propose employing the unscented Kalman filter (UKF) for handling the nonlinearities in 3D tracking, utilizing range and range-rate measurements. Additionally, we incorporate the probabilistic data association (PDA) algorithm with the UKF to handle cluttered environments. Through extensive simulations, we demonstrate a successful implementation of the UKF-PDA framework, showing that the proposed method effectively reduces bias and significantly advances tracking capabilities in passive bistatic radars.

## 1. Introduction

The reliable and accurate tracking of targets is an important problem in civil, industrial, and military applications. Most existing tracking systems employ active radar (or LIDAR or sonar) to obtain remote measurements on a target which are then fed into a tracking system. Active sensors have several undesirable properties, such as using spectral resources, creating emissions which can interfere with other systems, and providing emitter location information which can be exploited by an enemy. To overcome the disadvantages of active (cooperative) emissions, there is a rapidly increasing interest in using ambient (non-cooperative) environmental radiation in so-called passive radar to locate and track targets. The idea is very straightforward. Target reflections from transmissions such as AM and FM radio, digital free to air TV, cell phone base station transmissions (GSM etc.), emissions associated with navigation satellite systems, and a host of other sources [1,2,3] are harnessed to observe and track targets.

Passive radar is considerably less expensive than active radar because it does not use any high-power transmitter hardware. It is also spectrally efficient and capable of covert operation because it produces no emissions. However, the signal processing for high-performance detection and tracking can be challenging [4,5] because they rely on both direct and reflected electromagnetic signal from a wide range of non-cooperative sources operating across a possibly wide frequency band. Target information such as range and Doppler are derived from estimated differences between a direct signal and a reflected version of the same signal. Bearing can be estimated given an appropriate antenna, but this is often a challenging problem, and bearing estimates can be very unreliable [6,7]. Digital television (DTV) broadcasts are a potentially good choice of illuminator of opportunity for passive radar because of their high power and wide coverage [8].

Target tracking is a broad term encompassing a set of algorithms designed to detect and track moving objects based on sequential measurements obtained from sensors. It involves several critical processes including track initiation and termination (detecting the appearance and disappearance of tracks based on sensor measurements, often referred to as track maintenance), data association (assigning sensor measurements to specific tracks or identifying them as clutter or false measurements), and track smoothing (fitting feasible track paths through sequences of noisy sensor measurements associated with a particular track). Sensor measurements can exhibit linear or nonlinear relationships with track kinematic variables, and they are susceptible to noise, false measurements, and missed true measurements. Track kinematic variables can evolve linearly or non-linearly, often comprising linear sections and maneuvers.

Early tracking systems employed M-out-of-N sequential detectors for track initiation and termination, nearest-neighbor (NN) algorithms and gating for data association, as well as recursive least-squares alpha-beta filters with maneuver detection gates for track smoothing. Although these methods performed well in many scenarios, they faced limitations in dense multiple target scenarios, environments with heavy clutter, highly maneuvering targets, and strongly nonlinear sensor models. Consequently, research efforts over the past 50 years have aimed to overcome these limitations and integrate these processes (track maintenance, data association, track smoothing) into computationally feasible recursive algorithms capable of accurate tracking under various sensor models and track evolution scenarios.

The integration of these tracking processes began in 1964 with Sittler’s work [9] and continued with the introduction of the probabilistic data association (PDA) algorithm by Bar-Shalom in 1973 [10]. The PDA algorithm integrated data association with track smoothing in cluttered environments and was subsequently extended to handle multiple targets through joint PDA (JPDA). In 1979, Reid [11] proposed multiple hypothesis tracking (MHT), which theoretically integrated all tracking processes but was constrained by its high computational load. The use of the Kalman filter for track smoothing, both for maneuvering and non-maneuvering targets, gained traction after Singer’s 1971 paper [12], followed by the interacting multiple model filter (IMM) introduced by Blum and Bar-Shalom in 1988 [13]. The early 1990s saw the introduction of the integrated PDA (iPDA) algorithm by Musicki and Evans [14], which combined track maintenance with PDA, and later the joint iPDA (JiPDA). Around the same time, Gordon’s particle filter [15] provided an effective alternative to the extended Kalman filter (EKF) for nonlinear sensor and track models, followed by the unscented Kalman filter (UKF) in 2000 [16]. There have been further developments in tracking techniques, although we will not cover them in detail here. The tracking literature is replete with acronyms denoting the various techniques employed to handle the fundamental tracking processes, such as UKF-PDA, UKF-JPDA-IMM, etc. Notably, a recent example of a comprehensive tracking filter is the particle Metropolis–Hastings multi-target tracker published by Vu, Vo, and Evans in 2014 [17], representing an advanced version of a full MHT tracker.

In this paper, we study target tracking in a Cartesian framework using only range and range-rate information derived from a bistatic passive radar. The relationship between the target state and the measurements is non-linear, which further complicates the tracking problem. Standard tracking filters based on the extended Kalman filter (EKF) perform poorly in such circumstances, resulting in unstable and biased track estimates, even when bearing measurements are available, although less so [18,19,20]. To overcome the problem of bias when using only range and range rate measurements, we develop a tracker based on an unscented Kalman filter (UKF). The UKF deals with the nonlinear transformation between target states and target measurements by propagating forward multiple points from the state covariance matrix and determining a more accurate state covariance matrix, rather than simply propagating the mean and covariance based on linearization as is performed in the EKF [16]. We employ a standard Poisson distribution to model clutter measurements accurately. However, in future work, we plan to incorporate track initiation and termination techniques inspired by integrated probabilistic data association (IPDA) [14], to automatically initiate and terminate tracks based on a track life model. This paper focuses on addressing the challenges posed by a nonlinear measurement model from a bistatic passive sensor and clutter. To efficiently handle this, we examine the computational efficiency of an UKF-PDA combination. Our future work will extend these findings to include track maintenance and tracking of strongly maneuvering targets using the IMM-UKF-JiPDA approach. The results and insights gained from this research will be presented in a forthcoming paper, building upon the foundations established in this study.

The main contributions of this paper are:

- Improved tracking accuracy by eliminating bias in 3D Cartesian coordinates using range and range-rate measurements without angular information.
- Development of a framework for utilizing the extended Kalman filter (EKF) in passive radar systems without requiring knowledge of bearing angles.
- Development of a framework for applying the unscented Kalman filter (UKF) in passive radar environments without the need for bearing angle information.
- Integration of probabilistic data association (PDA) with the unscented Kalman filter (UKF) for low bias tracking in cluttered bistatic passive radar environments, considering a probability of detection less than one and accommodating occasional missed detections.
- Performance analysis of the proposed system design and framework, including the integration of UKF and PDA.

To the best of the author’s knowledge, there exist no solutions for integrating an EKF and UKF, and with the inclusion of PDA for passive bistatic radar-based tracking and hence, we consider the work presented here to be quite novel.

## 2. Literature Review

When there is a nonlinear relationship between the state and the measurement, the standard and commonly used algorithm for target tracking and localization is the extended Kalman filter (EKF) [21,22], which was initially developed for the Apollo Mission [23,24]. Extended Kalman filtering had been a huge success for applications in which the errors introduced by linear approximations are insignificant compared to the errors due to measurement noise. For example, every global navigation satellite system (GNSS) receiver uses an EKF to estimate its own position and velocity and to synchronize the receiver clock with GPS time [25]. However, there are well-known cases where the errors introduced by the linearization are significant, and this makes the EKF a sub-optimal and biased estimator [26,27]. In order to address this problem, various filtering strategies have been developed, including sigma-point filters [28], unscented Kalman filters (UKF) [27], particle filters [15] and many more.

A particle filter [15] uses the Monte Carlo sampling method to calculate the posterior density of the tracks given measurements via the Bayes rule. Although in principle, this approach can handle any nonlinear system if the appropriate probability density functions are known, computation can become excessive even with Monte Carlo sampling.

UKF methods have proven to be superior to EKF filters in a wide range of applications [29]. UKF uses the so-called unscented transform in which deterministic sample points cater for nonlinearities of the systems [26]. Furthermore, UKF works without calculating the Jacobian matrix and still gives better performance. However, it has no general formula for calculating the values of the sigma points. Hence, it is necessary to make correct approximations of the parameters depending on the tracking setup. Otherwise, there is a chance of poor tracking performance. The study referenced in [30] utilizes EKF and UKF algorithms for tracking in both Gaussian and non-Gaussian noise conditions. The author notes that the EKF demonstrates reliable performance in a narrower set of circumstances and exhibits greater unpredictability compared to the UKF. Overall, the UKF is usually the more consistent of the two. In another research [31], bearings only tracking estimation of an autonomous underwater vehicles in the presence of unknown sensor position is investigated using UKF. The tracking performance of both UKF and EKF is precisely compared. Simulation results show that an UKF-based filter has better tracking performance in comparison to EKF in both ideal and realistic scenarios.

Application of an unscented Kalman filter in any system is not trivial. In [27], the author describes the general unscented transform (UT) mechanism along with a variety of special formulations that can be tailored to the specific requirements of different nonlinear filtering and control applications.

Another challenge of tracking in passive radar is the measurement model. A traditional radar system assumes the bearing information is available in the measurement model [32,33,34]. Similarly, in [32], UKF has been applied to FM passive bistatic radar having a measurement model made up of bistatic range, azimuth, elevation, and bistatic Doppler frequency. In our case, we assume no bearing information to perform tracking considering a scenario similar to [35], where the author has tracked multiple targets in a passive bistatic radar setting using range and range-rate measurements. However, the focal point of the research in [35] is track initialization of multidimensional assignment problem in multistatic environment using EKF.

When tracking targets that have a probability of detection below one in the presence of false alarms (also known as clutter), it is crucial to make informed decisions about which measurements to use for updating each track. Many algorithms have been created to tackle this issue, as noted in sources such as [36,37,38]. Among these algorithms, two simpler options include the strongest-neighbor filter (SNF) and the nearest-neighbor filter (NNF).

The strongest-neighbor filter (SNF) selects the validated measurement with the highest intensity within the validation gate for track updating, discarding the rest of the measurements. On the other hand, the nearest-neighbor filter (NNF) chooses the measurement closest to the predicted measurement to update the track. Although these simple algorithms can work well in low-clutter situations, they become less effective as the probability of false alarms increases, such as in the case of low observable maneuvering targets [39,40]. An alternative approach is to use all validated measurements with different weights or probabilities, rather than choosing only one measurement from the received set. This approach is called probabilistic data association (PDA) [41]. Standard PDA and its various improved versions have proven to be highly effective in tracking a single target in clutter [42,43]. In our paper, we utilize the combination of probabilistic data association and the unscented Kalman filter in bistatic passive radar environment. To the best of our knowledge, there are no other papers that have implemented this approach.

In [40], the author used a probability hypothesis density (PHD) filter in a 2D tracking scenario for a passive multi-static radar system (PMR) that utilizes multiple FM transmitters and a single receiver. In this contribution, our interest lies in three-dimensional tracking and dealing with clutter.

## 3. System Model

We consider a constant velocity (CV), constant heading target model in three-dimensional Cartesian space. For the CV model, the target’s location is $p\left(t\right)={\left[x\left(t\right)y\left(t\right)z\left(t\right)\right]}^{\tau }$ and the target’s velocity is $v\left(t\right)={\left[\dot{x}\left(t\right)\dot{y}\left(t\right)\dot{z}\left(t\right)\right]}^{\tau }$. Here, t=1,2,⋯,T is the time index. The kinematic equation for target’s motion is given by,

(1) $$x\left(t+1\right)=Fx\left(t\right)+\eta \left(t\right)$$

where the state vector $x\left(t\right)$ is given by,

(2) $$x\left(t\right)=\left[\begin{array}{c} p\left(t\right)\\ v\left(t\right) \end{array}\right]$$

and $F=I_{3}⊗\tilde{F}$ is system state transition matrix, ⊗ is Kronecker product and $I_{3}$ is a 3 × 3 identity matrix used to project the motion of the target in all three axis of the three-dimensional Cartesian space. $\eta \left(t\right)$ is zero-mean white Gaussian noise whose covariance matrix is $Q=\sigma_{p}^{2}I_{3}⊗\tilde{Q}$, where $\sigma_{p}^{2}$ is the process noise of the system. For the CV model,

(3) $$\tilde{F}=\left[\begin{array}{cc} 1 & \Delta T\\ 0 & 1 \end{array}\right]$$

and

(4) $$\tilde{Q}=\left[\begin{array}{cc} \Delta T^{4}/4 & \Delta T^{3}/2\\ \Delta T^{3}/2 & \Delta T^{2} \end{array}\right]$$

where ΔT is the sampling interval.

**Figure 1:**
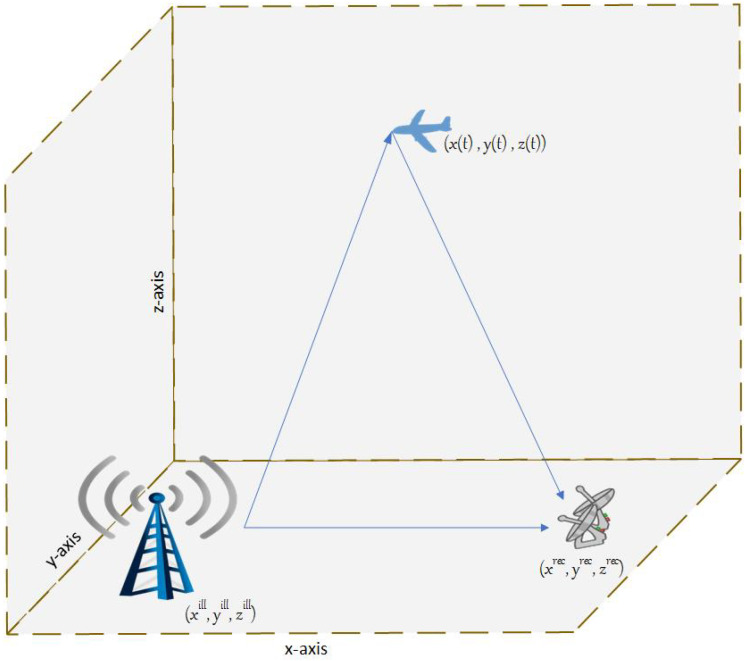
System model.

Assume a stationary receiver and a stationary illuminator are located at $p^{rec}={\left[x^{rec}y^{rec}z^{rec}\right]}^{\tau }$ and $p^{ill}={\left[x^{ill}y^{ill}z^{ill}\right]}^{\tau }$, respectively, as shown in Figure 1. We assume that the available measurements are in the form of bistatic range $\gamma \left(t\right)$ and range rate (Doppler shift) $\dot{\gamma }\left(t\right)$. Hence, the measurement vector z(t) is given by,

(5) $$z\left(t\right)=\left[\begin{array}{c} \gamma \left(t\right)\\ \dot{\gamma }\left(t\right) \end{array}\right]$$

The relationship between the elements of state vector $x\left(t\right)$ and measurement vector $z\left(t\right)$ is nonlinear, as can be seen in (7) and (8). Assume *h* is the nonlinear transformation of the state vector to the measurement vector. The mathematical expression of the nonlinear transformation can be written as,

(6) $$z\left(t\right)=h\left(x\left(t\right)\right)+w\left(t\right)$$

where $w\left(t\right)$ is the independent, zero-mean Gaussian measurement noise with covariance matrix $R\left(t\right)$,

$$R\left(t\right)=\left[\begin{array}{cc} \sigma_{\gamma }^{2} & 0\\ 0 & \sigma_{\dot{\gamma }}^{2} \end{array}\right]$$

where $\sigma_{\gamma }^{2}$ and $\sigma_{\dot{\gamma }}^{2}$ represents the measurement error variances of the bistatic range and range rate, respectively. The bistatic range and range rate measurements are given by

(7) $$\gamma \left(t\right)=\left|\right|p\left(t\right)-p^{ill}\left|\right|+\left|\right|p\left(t\right)-p^{rec}\left|\right|$$

(8) $$\dot{\gamma }\left(t\right)=-\frac{f}{c}\cdot \left[\frac{{\left(p\left(t\right)-p^{rec}\right)}^{\tau }}{\left|\right|p\left(t\right)-p^{rec}\left|\right|}+\frac{{\left(p\left(t\right)-p^{ill}\right)}^{\tau }}{\left|\right|p\left(t\right)-p^{ill}\left|\right|}\right]\cdot v\left(t\right)$$

here *f* is the illuminator’s carrier frequency and *c* is the speed of light.

In this paper, we develop and simulate an UKF-based tracker incorporating probabilistic data association (PDA) to handle clutter. In Section 4, below, we describe the development of track smoothing algorithm for both the extended Kalman filter (EKF) and the unscented Kalman filter (UKF). The inclusion of probabilistic data association with UKF is presented in Section 5.

## 4. Tracking of a Target Using Range and Range-Rate Measurements

In this section, we present the two methods that we study for tracking a target with a bistatic passive radar, given measurements are in the form of range and range-rate. The tracking methods are extended Kalman Filter (EKF) and the unscented Kalman Filter (UKF).

### 4.1. Extended Kalman Filter

In the extended Kalman filter, the nonlinear predicted measurement $\hat{z}\left(t+1|t\right)$ of the predicted state $\hat{x}\left(t+1|t\right)$ is expressed with the equation given below

(9) $$\hat{z}\left(t+1|t\right)=h\left(\hat{x}\left(t+1|t\right)\right)$$

where *h* is the nonlinear function of the state space mapping it to the measurement space. The Jacobian of *h* is calculated at the predicted state $\hat{x}\left(t+1|t\right)$, as it cannot be applied directly to the covariance update.

(10) $$H_{E}=\frac{\partial h}{\partial x}{|}_{x=\hat{x}\left(t+1|t\right)}$$

The measurement covariance $S_{E}$, Kalman gain $K_{E}$, and state estimate’s covariance update $P_{E}$ of the EKF are given by,

$$S_{E}\left(t+1\right)=R\left(t\right)+H_{E}P_{E}\left(t+1|t\right){H_{E}}^{\tau }$$

$$K_{E}\left(t+1\right)=P_{E}\left(t+1|t\right)\;{H_{E}}^{\tau }S_{E}{\left(t+1\right)}^{-1}$$

$$P_{E}\left(t+1|t+1\right)=\left(I-K_{E}\left(t+1\right)H_{E}\right)P_{E}\left(t+1|t\right)$$

The Jacobian of the nonlinear measurement function *h* mentioned in (10) for the EKF can be written as

(11) $$\begin{array}{c} H_{E}=\left[\begin{array}{cccccc} \frac{\partial \gamma }{dx} & \frac{\partial \gamma }{d\dot{x}} & \frac{\partial \gamma }{dy} & \frac{\partial \gamma }{d\dot{y}} & \frac{\partial \gamma }{dz} & \frac{\partial \gamma }{d\dot{z}}\\ \frac{\partial \dot{\gamma }}{dx} & \frac{\partial \dot{\gamma }}{d\dot{x}} & \frac{\partial \dot{\gamma }}{dy} & \frac{\partial \dot{\gamma }}{d\dot{y}} & \frac{\partial \dot{\gamma }}{dz} & \frac{\partial \dot{\gamma }}{d\dot{z}} \end{array}\right] \end{array}$$

where the derivatives of the bistatic range and range rate measurements given in (7) and (8), respectively, w.r.t *x*-axis are calculated as below:

(12) $$\begin{array}{cc} \frac{\partial \gamma }{dx}= & \frac{x\left(t\right)-x_{rec}}{\left|\right|p-p^{rec}\left|\right|}+\frac{x\left(t\right)-x^{ill}}{\left|\right|p-p^{ill}\left|\right|} \end{array}$$

(13) $$\begin{array}{cc} \frac{\partial \gamma }{d\dot{x}}= & 0\\ \frac{\partial \dot{\gamma }}{dx}= & \frac{x(t)}{\left|\right|p-p^{rec}\left|\right|}-\frac{\left(x\left(t\right)-x^{rec}\right)\zeta_{r}^{rec}}{\left|\right|p-p^{rec}{\left|\right|}^{3}} \end{array}$$

(14) $$\begin{array}{cc} \; & +\frac{x(t)}{\left|\right|p-p^{ill}\left|\right|}-\frac{\left(x\left(t\right)-x^{ill}\right)\zeta_{s}^{ill}}{\left|\right|p-p^{ill}{\left|\right|}^{3}} \end{array}$$

(15) $$\begin{array}{cc} \frac{\partial \dot{\gamma }}{d\dot{x}}= & \frac{x\left(t\right)-x^{rec}}{\left|\right|p-p^{rec}\left|\right|}+\frac{x\left(t\right)-x^{ill}}{\left|\right|p-p^{ill}\left|\right|} \end{array}$$

where

(16) $$\begin{array}{cc} \zeta_{r}^{rec}= & \left(x\left(t\right)-x^{rec}\right)\dot{x}\left(t\right)\;+\;\left(y\left(t\right)\;-\;y^{rec}\right)\dot{y}\left(t\right)\;+\left(z\left(t\right)\;-\;z^{rec}\right)\dot{z}\left(t\right)\; \end{array}$$

(17) $$\begin{array}{cc} \zeta_{s}^{ill}= & \left(x\left(t\right)-x^{ill}\right)\dot{x}\left(t\right)\;+\;\left(y\left(t\right)\;-\;y^{ill}\right)\dot{y}\left(t\right)\;+\left(z\left(t\right)\;-\;z^{ill}\right)\dot{z}\left(t\right)\; \end{array}$$

The derivatives of the bistatic range and range rate measurements w.r.t *y*-axis and *z*-axis can be calculated in the same manner as above.

### 4.2. Unscented Kalman Filter

**Figure 2:**
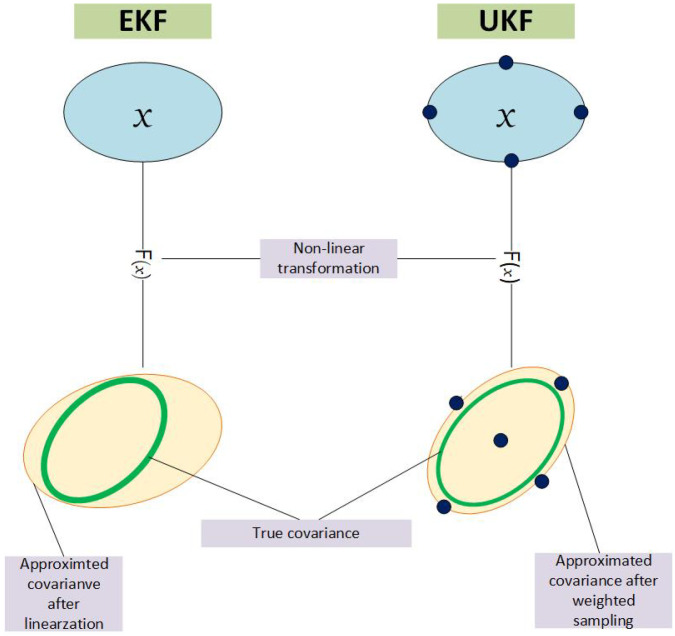
Illustration of EKF and UKF.

An unscented Kalman filter (UKF) [26] is implemented by using sampling points called sigma points. Instead of propagating only one point, which is the mean, this set of sigma points is propagated through the nonlinear function; as a result, a more accurate mean and covariance of the state is recovered. An illustration showing the transformation of state space to measurement space using EKF and UKF is presented in Figure 2. The black circles represent the sigma points in UKF transformation. The details of all the steps involved in applying the UKF algorithm are as follows.

#### 4.2.1. Calculation of Sigma Points

A set of 2n+1 sigma points is derived from the state x(t) and covariance P(t) where *n* is the dimension of the state vector. The scaling parameter $\lambda =\alpha^{2}\left(n+\kappa \right)-n\;\mathrm{where}\;\alpha ,\kappa >0$. If χ represents the sigma point, then all the sigma points can be calculated using the expressions given by,

$$\chi_{0,t}=\hat{x}\left(t\right)$$

(18) $$\chi_{i,t}=\hat{x}\left(t\right)+{\left[\sqrt{\left(n+\lambda )\times P(t\right)}\right]}_{i}$$

when i=1,2,3,…,n

(19) $$\chi_{i,t}=\hat{x}\left(t\right)-{\left[\sqrt{\left(n+\lambda )\times P(t\right)}\right]}_{i-n}$$

when i=n+1,n+2,…,2n.

Furthermore, ${\left[\sqrt{\left(n+\lambda )\times P(t\right)}\right]}_{i-n}$ is the *i*th column of the matrix square root of (n+λ)×P(t). The matrix square root is calculated using numerically efficient and stable method known as the Cholesky decomposition.

#### 4.2.2. Time Update

The sigma points are propagated through the transition function *f*.

$$\chi_{\left(i,t+1|t\right)}=f\left(\chi_{i,t}\right)$$

The next step is to compute the predicted state mean $\hat{x}\left(t+1|t\right)$ and predicted covariance $\hat{P}\left(t+1|t\right)$ using weighted sigma points:

$$\begin{array}{cc} \hat{x}\left(t+1|t\right)= & \sum_{i=0}^{2n}w_{i}^{m}\chi_{\left(i,t+1|t\right)}\\ \hat{P}\left(t+1|t\right)= & \sum_{i=0}^{2n}w_{i}^{c}\left[\chi_{\left(i,t+1|t\right)}-\hat{x}\left(t+1|t\right)\right]\times {\left[\chi_{\left(i,t+1|t\right)}-\hat{x}\left(t+1|t\right)\right]}^{\tau }+Q_{t} \end{array}$$

where

(20) $$\begin{array}{cc} w_{0}^{m}= & \left[\frac{\lambda }{n+\lambda }\right] \end{array}$$

(21) $$\begin{array}{cc} w_{0}^{c}= & \frac{\lambda }{\left(n+\lambda \right)+\left(1-\alpha^{2}+\beta \right)} \end{array}$$

(22) $$\begin{array}{cc} w_{i}^{m}= & w_{i}^{c}=\frac{1}{2(n+\lambda )} \end{array}$$

with β>0 as a scaling parameter.

#### 4.2.3. Measurement Update

The subsequent step involves the transformation of sigma points into measurement space. If ψ represents the transformed sigma points in measurement space and is expressed as follows,

$$\psi_{\left(i,t+1|t\right)}=h\left(\chi_{\left(i,t+1|t\right)}\right)$$

the calculation of predicted mean of the measurement $\hat{z}\left(t+1|t\right)$ and covariance of the measurement $\hat{S}\left(t+1|t\right)$, and the cross-covariance of the state and measurement $\hat{C}\left(t+1|t\right)$ can be performed by using the following equations.

(23) $$\hat{z}\left(t+1|t\right)=\sum_{i=0}^{2n}w_{i}^{m}\psi_{\left(i,t+1|t\right)}$$

(24) $$\begin{array}{cc} \hat{S}\left(t+1|t\right)= & \sum_{i=0}^{2n}w_{i}^{c}\left[\psi_{\left(i,t+1|t\right)}-\hat{z}\left(t+1|t\right)\right]\times {\left[\psi_{\left(i,t+1|t\right)}-\hat{z}\left(t+1|t\right)\right]}^{\tau } \end{array}$$

(25) $$\begin{array}{cc} \hat{C}\left(t+1|t\right)= & \sum_{i=0}^{2n}w_{i}^{c}\left[\chi_{\left(i,t+1|t\right)}-\hat{x}\left(t+1|t\right)\right]\times {\left[\psi_{\left(i,t+1|t\right)}-\hat{z}\left(t+1|t\right)\right]}^{\tau } \end{array}$$

#### 4.2.4. State Update

Computation of the filter gain $K_{t+1}$, the updated state mean $\hat{x}\left(t+1|t+1\right)$, and updated covariance $\hat{P}\left(t+1|t+1\right)$ is conducted with the following equations,

(26) $$K_{t+1}=\hat{C}\left(t+1|t\right)\times {\hat{S}\left(t+1|t\right)}^{-1}$$

(27) $$\hat{x}\left(t+1|t+1\right)=\hat{x}\left(t+1|t\right)+K_{t+1}\left[z\left(t+1\right)-\hat{z}\left(t+1|t\right)\right]$$

(28) $$\hat{P}\left(t+1|t+1\right)=\hat{P}\left(t+1|t\right)-K_{t+1}\hat{S}\left(t+1|t\right)K_{t+1}^{\tau }$$

### 4.3. Numerical Instability

The unscented Kalman filter is susceptible to numerical instability, and a common issue arises when the noise covariance is small. In such cases, rounding errors may lead to the computation of a small positive eigenvalue as a negative number, causing the numerical representation of the state covariance matrix to become indefinite, whereas its true form is positive and definite. Positive-definite matrices can be efficiently computed using the Cholesky factorization algorithm. It is crucial to maintain the covariance matrix in this form to prevent it from acquiring a negative diagonal or becoming asymmetric.

Another instability occurs while computing and updating the state co-variance matrix. The covariance matrix is expected to be symmetric, positive, and definite, but for some updates it fails to be so; hence, it is required to approximate it to the nearest positive-definite matrix. This problem can be solved by using nearest symmetric positive semi-definite matrix given in [44]. To the best of the author’s knowledge, this technique does not appear to have been used with the unscented Kalman filter in the previous literature. However, our result shows that it is effective with the unscented Kalman filter.

## 5. Probabilistic Data Association

In this section, we incorporate probabilistic data association (PDA) [41] into the UKF [26] framework for target tracking in the presence of clutter. The PDA algorithm differs from other methods as it does not solely rely on the most probable value of measurements. Instead, it calculates the association probabilities of each validated measurement to the target of interest at the current time, taking into account all validated measurements rather than relying on assumptions and rules. This probabilistic or Bayesian information is utilized in the probabilistic data association filter (PDAF) algorithm, which accommodates for measurement uncertainty. It is worth noting that there is no literature to date that reports the use of PDA with the UKF in a bistatic passive radar environment.

**Figure 3:**
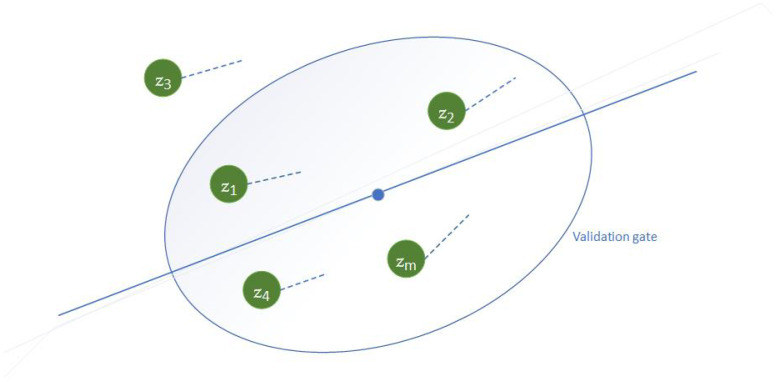
Measurement validation illustration.

The PDA algorithm is executed in three main steps which are measurement validation/gating phase, data association and state estimation. Validation of measurements is performed by the gating process. The gate is formed around the predicted measurement. Only the measurements within the gate are considered for association with the track. The size of the gate determines the probability of measurements falling within the gate which is called gate probability. The measurement validation phase is illustrated in Figure 3. A precise equation for the measurement validation in the ellipsoidal gating region is given by

(29) $$D_{i}{\left(t+1\right)}^{2}={\left(z_{i}\left(t+1\right)-\hat{z}\left(t+1|t\right)\right)}^{\tau }S{\left(t+1\right)}^{-1}\times \left(z_{i}\left(t+1\right)-\hat{z}\left(t+1|t\right)\right)<\nu^{2}$$

where ν is the gating threshold value corresponding to the gate probability $P_{G}$. Once measurement validation is conducted, the set of validated measurements satisfying (29) can be represented as

$$z\left(t+1|t\right)={\left\{z_{i}\left(t+1\right)\right\}}_{i=1}^{m}$$

where *m* is the number of valid measurements at time t+1. The overall PDA measurement estimate is given by a weighted combination of all the validated measurements given by the following equation

(30) $$\Upsilon \left(t+1\right)=\sum_{i=1}^{m}\beta_{i}\left(t+1\right)z_{i}\left(t+1\right)$$

where $\beta_{i}\left(t+1\right)$ is the association probability given by the likelihood function $q_{i}\left(t+1\right)$ given as

(31) $$\beta_{i}\left(t+1\right)=\frac{q_{i}\left(t+1\right)}{\sum_{i=0}^{m}q_{i}\left(t+1\right)}$$

where

$$\begin{array}{c} q_{i}\left(t+1\right)=\{\begin{array}{cc} \Lambda (1-P_{D}P_{G}) & i=0\\ \frac{\exp\left(-\frac{1}{2}D_{i}{\left(t+1\right)}^{2}\right)P_{D}}{{\left(2\pi \right)}^{c/2}{\left|S\left(t+1\right)\right|}^{1/2}}\; & i\neq 0 \end{array} \end{array}$$

In the above equation, Λ is the clutter density, *c* is the dimension of measurement vector, S(t+1) is defined in (24), $D_{i}{\left(t+1\right)}^{2}$ is defined in (29), $P_{D}$ is the probability of detection, and $P_{G}$ is the gate probability.

Once PDA measurement Υ(t+1) is calculated, the state update step can be expressed as:

(32) $$\hat{x}\left(t+1|t+1\right)=\hat{x}\left(t+1|t\right)+K_{t+1}\left[\Upsilon \left(t+1\right)-\hat{z}\left(t+1|t\right)\right]$$

A combined effect of all the original and clutter measurements expressed in the form of Υ(t+1) given in (32) is used to update the predicted state. Hence, by replacing (29) with (32), the tracking explained in Section 4.2 is applicable to the case of a cluttered environment via incorporation of PDA with UKF.

## 6. Simulation Studies

In this section, the performance of the proposed technique incorporating PDA with UKF is presented. The proposed system model was simulated in Matlab using a Monte Carlo approach based on the parametric values identified below.

The simulation scenario consists of a single stationary receiver and a single illuminator. The receiver is located at the origin of the coordinate system, and the illuminator is placed at [−2000m,−2000m,100m]. The carrier frequency of the transmitter is 400 MHz, and the scan period is 1 s. The system noise intensity is set at $\sigma_{p}=0.5$ m, and the target’s initial position and velocity are [−4000m,2000m,1000m] and [−37m/s,−36m/s,−17m/s], respectively. The entire tracking time is 40 scans.

To start the tracking process, an initial estimate of the target’s position and velocity is required. However, the track initialization is not the focus of this research, so we initiate the tracks by adding small errors to the true position and velocity of the target. The errors are $e_{1}=60\;m$ and $e_{2}=2.5\;m/s$, added to the true position $p_{0}$ and true velocity $v_{0}$, respectively. The measurement noise for range and range rate are set at $\sigma_{r}=50\;m$ and $\sigma_{\dot{r}}=5\;\mathrm{Hz}$, respectively.

**Figure 4:**
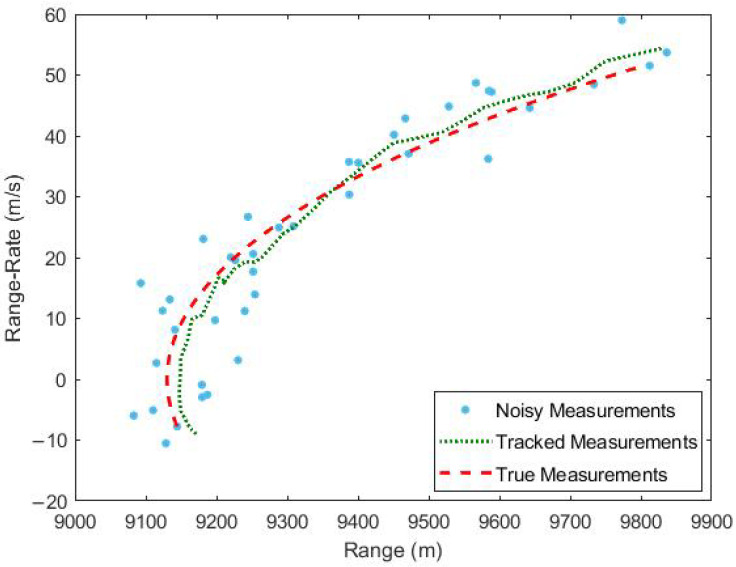
Tracking using EKF.

Once the simulation scenario is established, the subsequent step involves tracking the target via a traditional EKF. The actual and tracked measurements of the target are illustrated in Figure 4, and the input to the EKF comprises noisy range and range-rate measurements. The outcomes indicate that the EKF is capable of tracking the target in the measurement domain, specifically range and range-rate. Nevertheless, observing the tracking outcomes in three-dimensional Cartesian space is more valuable in real-world circumstances.

**Figure 5:**
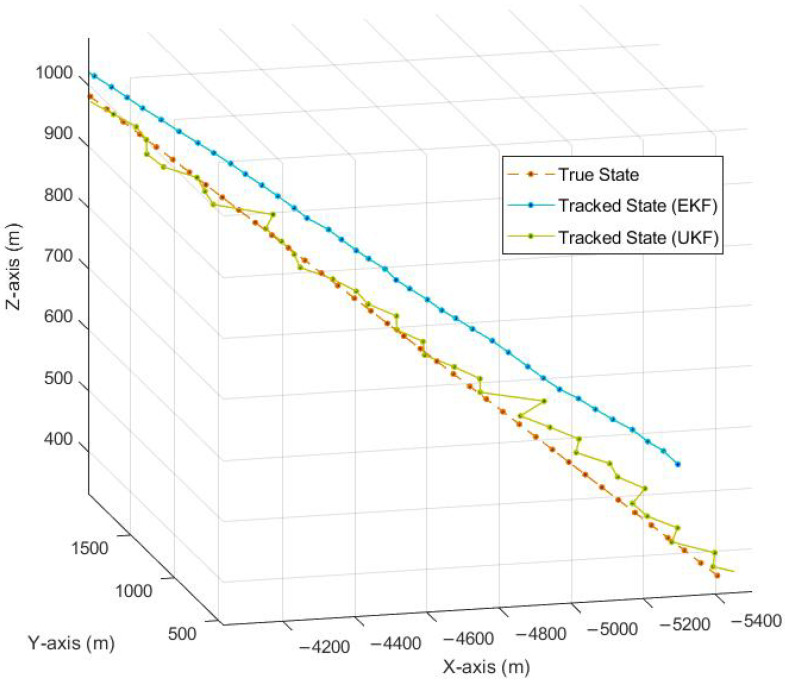
Tracking using EKF and UKF.

In order to transform the tracked measurements from the range and range-rate domain to the three-dimensional Cartesian domain, which is the actual requirement of the tracking system, the linearization of the measurements is conducted by using the Jacobian in (11). Once the Jacobian is calculated, tracking is performed using EKF and can be seen in Figure 5. We can see that the transformation of the coordinates generates a constant moderate bias along the z-axis. The observed bias increases if the initial position and velocity errors are high and decreases the tracking accuracy as a result. As a solution, to reduce this bias arising due to the nonlinear transformation of the state space model, an unscented Kalman filter (UKF) (explained in Section 4.2) is deployed to the system. Hence, in Figure 5, we can see a major reduction in bias when tracking is conducted with UKF. Here, the scaling parameters for the unscented Kalman filter are set to α=0.1,β=2 and κ=5−n, where n=6 is the size of the target state vector.

**Figure 6:**
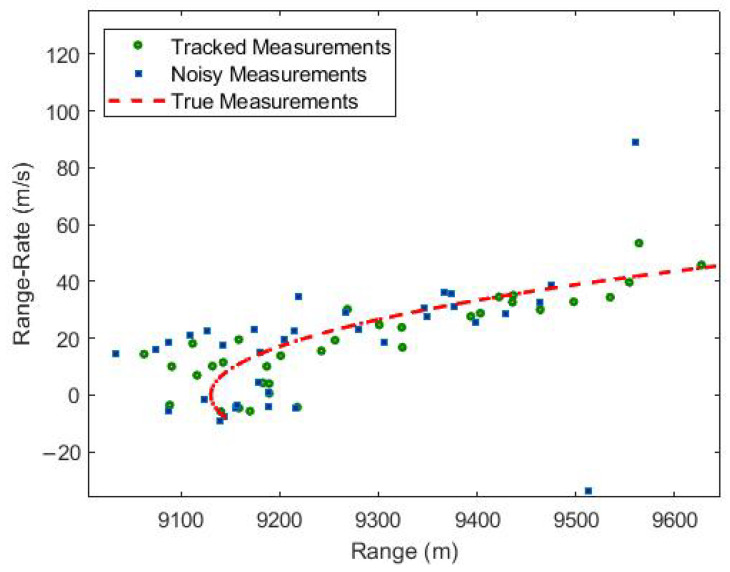
Tracking using UKF.

Additionally, the tracking results of the UKF algorithm in the range and range-rate domain are shown in Figure 6. This figure is included to ensure that the correction of bias in the Cartesian domain does not result in any unintended consequences in the range and range-rate domain. Figure 6 displays the tracking performance of UKF in the range-range rate domain. The figure clearly demonstrates that the tracked measurements closely match the true measurements in both the range and range-rate domain.

Further, in this study, the performance of an UKF in the presence of clutter is analyzed through simulations. The measurements from clutter and the measurements from the target are both used as input for the PDA algorithm that was discussed in Section 5. The number of clutter measurements is set to be Poisson distributed for simulation purposes and remains constant for the entire scanning duration. Validation of the measurements is carried out using Equation (29). The parameters used for the PDA algorithm in the simulations are listed in Table 1. $\Omega_{max}$ represents the largest eigenvalue of the measurement covariance matrix S.

**Figure 7:**
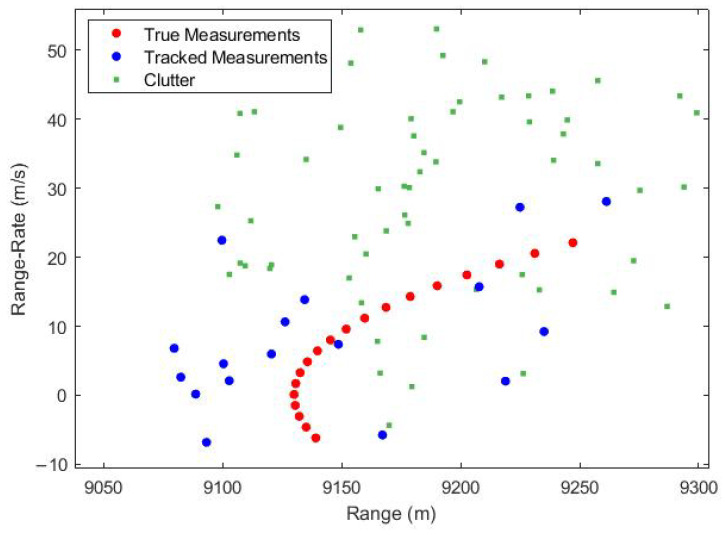
Tracking using UKF and PDA in Range and Range-rate domain.

**Figure 8:**
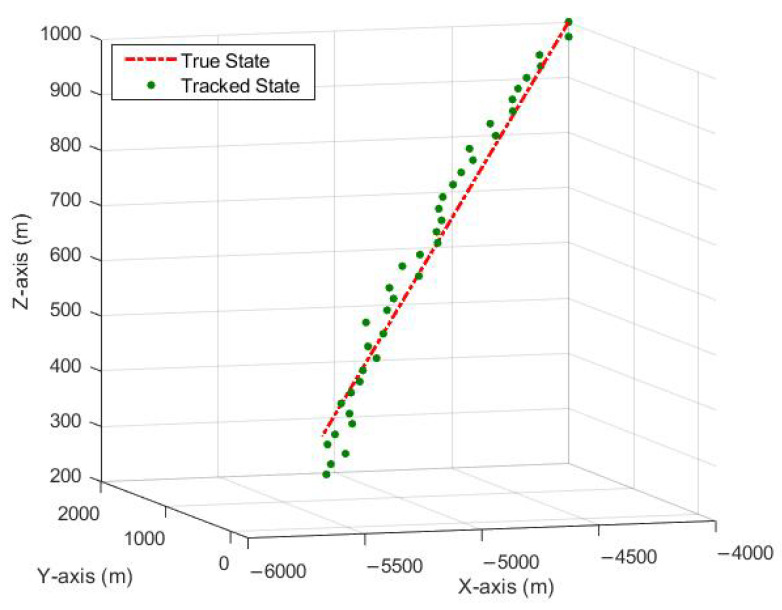
Tracking using UKF and PDA in 3D Cartesian Domain.

It can be seen from Figure 7, that the tracked measurements converge towards the true measurements. In other words, the UKF with PDA is successfully tracking the target in the range and range-rate domain. It can be observed from the results in Figure 8 that the proposed technique of incorporating PDA with UKF successfully tracks the target in a cluttered environment without noticeable bias. The integration of PDA provides the UKF with optimized measurements, thereby reducing the measurement noise and improving the overall performance of the tracking system.

**Figure 9:**
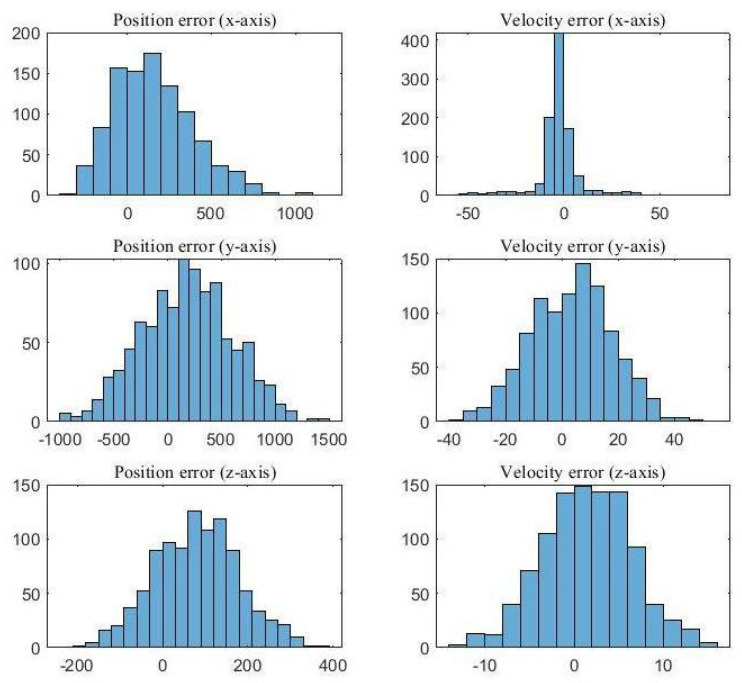
Histogram of the tracker at Scan 35.

The effectiveness of the UKF-PDA framework in handling cluttered environments is further emphasized in Figure 9. This figure illustrates histograms of position and velocity errors on all axes at scan number 35, which have been computed using the root mean square error (RMSE) method. Originating from 1000 Monte Carlo simulations, the majority of these histograms showcase errors with a mean value close to zero, thus highlighting the UKF-PDA’s efficacy in reducing bias, a common concern with the conventional EKF tracking approach. Overall, these findings underscore the robustness and reliability of the UKF-PDA framework in cluttered environments, leading to more accurate tracking outcomes.

**Figure 10:**
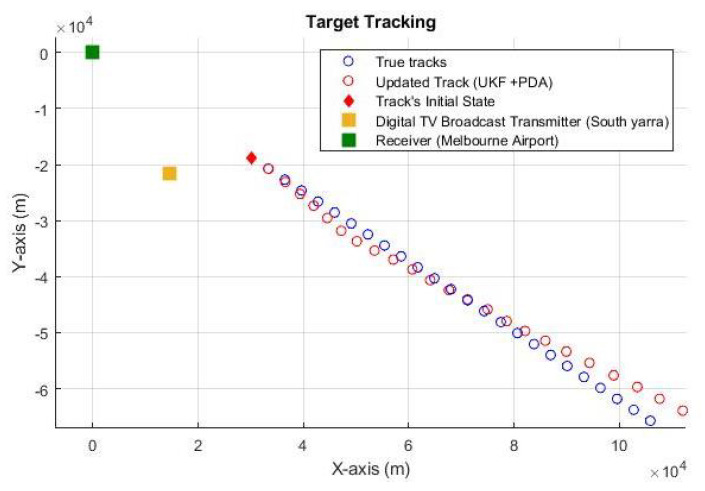
Tracking of the actual aircraft using real-time measurements.

In our simulation studies, we also employed real-time measurements obtained from one of the largest ADS-B networks worldwide, comprising over 35,000 connected receivers [45]. Our study focused on tracking a specific flight from Melbourne, Australia, to Hobart, Australia. To facilitate the tracking process, we utilized the South Yarra Digital TV Broadcast as the illuminator, transmitting at a frequency of 640 MHz with a power output of 85.00 W. We strategically positioned the receiver at Melbourne International Airport in Tullamarine, Australia. Furthermore, we skillfully utilized the Haversine formula to convert raw measurements, initially in longitude and latitude coordinates, into a more applicable format encompassing range and range-rate values. Figure 10 showcases the outcome of our analysis, demonstrating accurate tracking of the actual aircraft without significant bias. By integrating real-time measurements and specific flight details, we observed the precise tracking of the aircraft’s position and velocity. Additionally, it is important to note that our simulation studies considered the presence of clutter, caused by surrounding environmental conditions and other non-target objects. We took clutter effects into account to evaluate the robustness and accuracy of our tracking algorithm in real-world scenarios. The successful tracking results depicted in Figure 10 highlight the effectiveness of our approach in mitigating the impact of clutter and accurately estimating the target aircraft’s position and velocity during its flight from Hobart to Melbourne.

## 7. Conclusions

In summary, this study demonstrates the implementation of EKF and UKF algorithms in a passive bistatic radar system that lacks bearing angle information. The target is tracked in three-dimensional Cartesian space utilizing bistatic range and range rate measurements. The results indicate that the UKF algorithm outperforms the EKF in terms of tracking accuracy. The numerical issues encountered in the UKF are mitigated by utilizing approximations. Furthermore, the integration of the PDA algorithm demonstrates the UKF’s ability to work effectively in a cluttered environment. Despite numerical instability challenges, the successful implementation of the UKF-PDA framework significantly advances tracking techniques in passive bistatic radar, particularly in situations where reliable bearing information is unavailable or limited.

## Figures and Tables

**Table 1:** Parameters used in the PDA algorithm.

Parameter	Value
λ	4
$P_{d}$	0.9
$P_{G}$	1
ν	1.5 $\times \;\Omega_{max}$

## Data Availability

Available online at www.flightradar24.com.

## References

[1] Griffiths H.D., Baker C.J. (2005). Passive coherent location radar systems. Part 1: Performance prediction. IEE Proc. Radar Sonar Navig..

[2] Malanowski M., Kulpa K. Digital beamforming for Passive Coherent Location radar. Proceedings of the 2008 IEEE Radar Conference.

[3] Berger C., Demissie B., Heckenbach J., Willett P., Zhou S. (2010). Signal Processing for Passive Radar Using OFDM Waveforms. Sel. Top. Signal Process. IEEE J..

[4] Kuschel H., Heckenbach J., Muller S., Appel R. On the potentials of passive, multistatic, low frequency radars to counter stealth and detect low flying targets. Proceedings of the 2008 IEEE Radar Conference.

[5] Willis N.J. (2007). Advances in Bistatic Radar.

[6] Tharmarasa R., Kirubarajan T., McDonald M. Passive multitarget tracking using transmitters of opportunity. Proceedings of the 2009 IEEE Symposium on Computational Intelligence for Security and Defense Applications.

[7] Poullin D., Flecheux M. (2012). Passive 3D tracking of low altitude targets using DVB (SFN Broadcasters). IEEE Aerosp. Electron. Syst. Mag..

[8] Reimers U. (2008). DVB—Digitale Fernsehtechnik: Datenkompression und Übertragung.

[9] Sittler R.W. (1964). An Optimal Data Association Problem in Surveillance Theory. IEEE Trans. Mil. Electron..

[10] Bar-Shalom R. (1973). Tracking in clutter with probabilistic data association. IEEE Trans. Aerosp. Electron. Syst..

[11] Reid D.B. (1979). An algorithm for tracking multiple targets. IEEE Trans. Autom. Control..

[12] Singer R.A. (1971). Estimating optimal tracking filter performance for manned maneuvering targets. IEEE Trans. Aerosp. Electron. Syst..

[13] Blum R.S., Bar-Shalom Y. (1988). An interactive multiple model algorithm for systems with Markovian switching coefficients. IEEE Trans. Autom. Control.

[14] Musicki D., Evans R., Stankovic S. (1994). Integrated probabilistic data association. IEEE Trans. Autom. Control.

[15] Arulampalam M.S., Maskell S., Gordon N., Clapp T. (2002). A tutorial on particle filters for online nonlinear/non-Gaussian Bayesian tracking. IEEE Trans. Signal Process..

[16] Wan E.A., Van Der Merwe R. The unscented Kalman filter for nonlinear estimation. Proceedings of the IEEE 2000 Adaptive Systems for Signal Processing, Communications, and Control Symposium (Cat. No. 00EX373).

[17] Vu T.H.N., Vo B., Evans R. (2014). Particle Metropolis-Hasting multi-target tracker. IEEE Trans. Signal Process..

[18] Howland P.F. (1999). Target tracking using television-based bistatic radar. IEE Proc. Radar Sonar Navig..

[19] Nardone S., Lindgren A., Gong K. (1984). Fundamental properties and performance of conventional bearings-only target motion analysis. IEEE Trans. Autom. Control.

[20] Aidala V.J. (1979). Kalman filter behavior in bearings-only tracking applications. IEEE Trans. Aerosp. Electron. Syst..

[21] Corporation T., Gelb A. (1974). Applied Optimal Estimation.

[22] Bar-Shalom Y., Li X.R., Kirubarajan T. (2004). Estimation with Applications to Tracking and Navigation: Theory Algorithms and Software.

[23] Grewal M.S., Andrews A.P. (2010). Applications of Kalman filtering in aerospace 1960 to the present [historical perspectives]. IEEE Control Syst. Mag..

[24] Schmidt S.F. (1981). The Kalman filter-Its recognition and development for aerospace applications. J. Guid. Control.

[25] Grewal M.S., Weill L.R., Andrews A.P. (2007). Global Positioning Systems, Inertial Navigation, and Integration.

[26] Julier S.J., Uhlmann J.K. New extension of the Kalman filter to nonlinear systems. Proceedings of the Signal Processing, Sensor Fusion, and Target Recognition VI.

[27] Julier S.J., Uhlmann J.K. (2004). Unscented filtering and nonlinear estimation. Proc. IEEE.

[28] Van der Merwe R., Wan E. Gaussian mixture sigma-point particle filters for sequential probabilistic inference in dynamic state-space models. Proceedings of the 2003 IEEE International Conference on Acoustics, Speech, and Signal Processing, 2003 Proceedings, (ICASSP ’03).

[29] Van Der Merwe R. (2004). Sigma-Point Kalman Filters for Probabilistic Inference in Dynamic State-Space Models.

[30] Jahan K., Koteswara Rao S. (2020). Implementation Of underwater target tracking techniques for Gaussian and non-Gaussian environments. Comput. Electr. Eng..

[31] Costanzi R., Fenucci D., Manzari V., Caiti A. (2017). Bearing-only AUV tracking performance: Unscented Kalman Filter estimation against uncertainty in underwater nodes position. IFAC-PapersOnLine.

[32] Ortenzi L., Timmoneri L., Vigilante D. Unscented Kalman Filter (UKF) applied to FM band passive radar. Proceedings of the 2009 International Radar Conference “Surveillance for a Safer World” (RADAR 2009).

[33] Duan Z., Li X., Han C., Zhu H. Sequential unscented Kalman filter for radar target tracking with range rate measurements. Proceedings of the 2005 7th International Conference on Information Fusion.

[34] Bi S., Ren X.Y. (2008). Maneuvering target doppler-bearing tracking with signal time delay using interacting multiple model algorithms. Prog. Electromagn. Res..

[35] Bozdogan A.O., Soysal G., Efe M. Multistatic tracking using bistatic range—Range rate measurements. Proceedings of the 2009 12th International Conference on Information Fusion.

[36] Fortmann T., Bar-Shalom Y., Scheffe M. (1983). Sonar tracking of multiple targets using joint probabilistic data association. IEEE J. Ocean. Eng..

[37] Bar-Shalom Y., Tse E. (1975). Tracking in a cluttered environment with probabilistic data association. Automatica.

[38] Blackman S., Popoli R. (1999). Design and Analysis of Modern Tracking Systems.

[39] De Feo M., Graziano A., Miglioli R., Farina A. (1997). IMMJPDA versus MHT and Kalman filter with NN correlation: Performance comparison. IEE Proc. Radar Sonar Navig..

[40] Tobias M., Lanterman A.D. (2005). Probability hypothesis density-based multitarget tracking with bistatic range and Doppler observations. IEE Proc. Radar Sonar Navig..

[41] Chen J., Leung H., Lo T., Litva J., Blanchette M. (1996). A modified probabilistic data association filter in a real clutter environment. IEEE Trans. Aerosp. Electron. Syst..

[42] Lerro D., Bar-Shalom Y. (1993). Interacting multiple model tracking with target amplitude feature. IEEE Trans. Aerosp. Electron. Syst..

[43] Kirubarajan T., Bar-Shalom Y. (1996). Target motion analysis in clutter for passive sonar using amplitude information. IEEE Trans. Aerosp. Electron. Syst..

[44] Higham N.J. (1988). Computing a nearest symmetric positive semidefinite matrix. Linear Algebra Its Appl..

[45] FlightRadar24. https://www.flightradar24.com.

